# Professor Sunetra Gupta

**DOI:** 10.1192/bjb.2024.95

**Published:** 2025-04

**Authors:** Abdi Sanati

**Figure:**
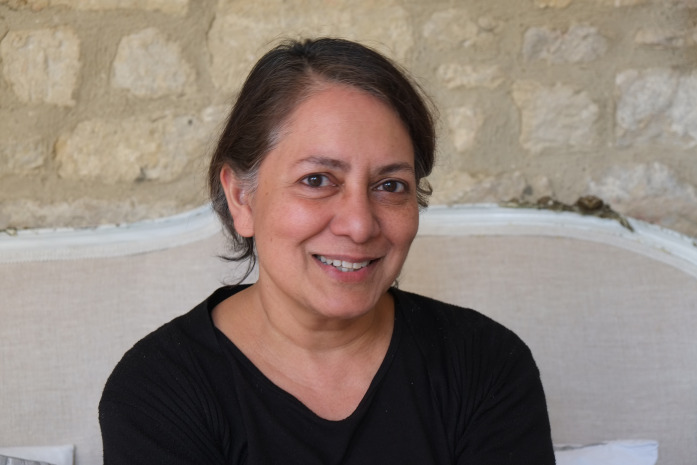

**The first question I have is what is your opinion on negative impacts of the COVID-19 lockdowns?**

My argument at the outset was that we can be very confident that lockdown could cause very serious harm. How can you deny the fact that a child who gets their only meal of the day at school would not be harmed when they were not allowed to go to school? You know about psychological harm much more than I do. I come from India, where closing down society means people die. The same could be argued for the rest of the Global South. Hundreds of millions of people have been pushed into poverty because of lockdowns. In 2020 we already had very good evidence that lockdowns cause harm. We have set up a charity called Collateral Global (collateralglobal.org), which attempts to document these harms. We did that because it seemed to me that in 2020, the harmful effect of lockdowns was by and large unacknowledged. My fundamental position on lockdowns is that they are extremely harmful. They selectively harm the poor and the young. I will not accept ignorance as an excuse. Where there was uncertainty was around the benefits of lockdown. We had certain harm balanced by uncertain benefits. I think what was done was exactly the opposite of the precautionary principle. We should have thought of different solutions to the problem rather than locking down.

**It's interesting you mentioned evidence. I've seen some recent research on the effectiveness of lockdowns which pointed to small effect size, if any. Why do you think that this evidence is not discussed much now?**

We didn't know in 2020 whether lockdowns stop spread. That uncertainty was balanced by the certainty that lockdowns would cause harm. The harms were obvious. The benefits were uncertain. But even if we agree with people who stated that it would stop the spread, there are other questions. What would stopping the spread achieve? One can make an argument on stopping the spread until a vaccine was developed for the vulnerable. But now it has become clear that lockdowns did not stop the spread, not in any measurable way. So you would think those people who are pro lockdowns would accept their error. You would think they would have the humility and the grace to say so. Not just because that's what you do as a scientist when you're wrong. But because the next time this happens, we need to make sure we don't repeat the same mistakes. So I am really astonished that that whole community are refusing to acknowledge that and insisting that we should have locked down earlier and harder.

**I have seen the serious negative effects of lockdown on people with severe mental illness and especially people with autism spectrum disorder. Do you think these harms would be considered in future pandemics?**

All we could do is set up an archive, and this we have done with our charity Collateral Global. It simply documents what has happened in various settings around the world without any judgements. All one can hope for is that there will be a growing slow realisation that lockdowns are a very inappropriate tool for dealing with the problem. I've previously likened it to taking a hammer to kill a fly sitting on a pane of glass. There is nothing we can do other than keep that record alive. The charity is doing pretty well. It has raised a lot of money and the director has published extensively on the harms of lockdown. It is there in the academic literature. Hopefully, people will realise that it was not inevitable and the costs far outweigh any benefits.

**Do you think the UK COVID-19 inquiry will ever address the question of lockdown?**

No. They have tried to address it. They asked me for a witness statement, which I provided. Then they didn't invite me to the stand, which is fine. But it is very disconcerting that they posed questions about my ideas. I mean literally reading off transcripts that I had provided to the inquiry itself and asking other people to respond to what I had said, without giving me the opportunity to do so. I don't think they want to listen to what I have to say but my witness statement is there for anyone to see,^1^ as is my statement to the Cabinet in September 2020.^2^

**I saw a sort of schism among scientists, and what I always wondered was why one group of scientists was selected over others by politicians to be the designated experts. Is there a process for selecting them?**

I don't think there were many of us who were saying something different. There's a network of scientists who do what I do, which is mathematical modelling of infectious diseases. Almost all of them were networked into a sort of larger group who were advising the government. I think there were only a few people like myself, who were questioning the dogma of lockdown, as it had become.

**Talking about modelling, the policy to start the lockdown was, at least to some extent, based on computer modelling.**

I use mathematical models, which are equation-based. I use computers to help me solve them. The type of models that were mainly used, certainly in the UK, were computer simulations. I'm a bit of a purist. I think you have to be very cautious when you're using a computer simulation. However, I don't think that's the reason we had a problem. The computer simulations pretty much did what any simple, much cleaner, mathematical model would do. The problem was in the assumptions they made on the way the pandemic had spread (or not) in any substantive way through the population. They made the assumption that the infection fatality rate was extremely high. The difference between what we argued and what was coming out of Imperial [College London] and other places is that we emphasised that there could be no certainty in the knowledge of when the pandemic arrived and what its infection fatality rate was, whereas they were very confident that it had a fatality rate of 1%. So the difference was more in the assumptions. I do not think the fundamental assumptions they made were backed up by anything at that point, except for a few bits of data.

**I admit that I learned these things many years ago, but we are talking about science and the way the hypotheses are tested. They are tested in the real world, not in computer models.**

Models are not good for testing hypotheses. They are hypothesis generation tools. You have to test them with data. In the pandemic, certain models were published that were highly circular. For example, Imperial published a letter claiming that the computer models had saved 400 000 lives. This was based on a model that had said 450 000 people would die. They came up with a computer simulation that said that meant they saved 400 000 lives. That's not science.

**That's interesting. So is it justified, without having proper data, to use these models for public policy-making? Because it seems to me that's what happened.**

I think that is what happened. You have to understand what the models are actually saying. We published in medRxiv an alternative model (just a set of simple equations) which showed that the data we had on deaths at March 2020 was compatible with a number of different scenarios.^3^ So you could have the situation that Imperial had modelled, which assumed the epidemic had only just arrived and was killing 1% of anyone infected. It was equally possible to fit a model in which the epidemic arrived in December 2019 and the infection fatality rate was very low. On that basis we said what we needed to do was to determine how many people had already been exposed to the virus. We even set up, by the end of March, a neutralising antibody test for SARS-CoV-2 in my lab. But then everything went kind of haywire and we were only able to test some blood donor samples from Scotland, which indicated that it had not spread there yet, except in Glasgow where we recorded a seropositivity of 11%. Mathematical models are quite useful to guide our thoughts and to generate hypotheses. The problem is not the model. It's this idea that you can fit it to data and then claim that fit is the only fit, whereas actually there are other fits, which I think were much more realistic.

**As psychiatrists we do a lot of risk assessments and our risk assessments have very low positive predictive values. And maybe I'm too naive, because when I looked at the way things were run at that time, it very much reminded me of our work, where we have to intervene with a large number of people, many of whom are false positives.**

I think that the way the models were used was wrong. It suggested that we had huge certainty about what was going to happen. Whereas, in fact, we were completely uncertain about the dynamics of the pandemic and where we were within the course of it. We were uncertain whether lockdowns work or not. The only thing we were certain about was that lockdowns would cause harm.

**It's interesting because in my work we have a lot of uncertainty and unfortunately we conflate uncertainty and risk. There is a book by Frank Knight in 1921 about separation of uncertainty and risk.^4^ This mixing of uncertainty and risk is harming my profession, and it seems that it had an effect during that time too.**

It is true.

**There is a quote from Stephen Schneider on the double ethical bind scientists face: the choice between being honest and being effective.^5^ It made me really think that scientists are trusted as purveyors of truth. But when they move to becoming agents of social engineering, I find it extremely concerning. What is your opinion on that?**

First of all, the idea that scientists are purveyors of truth is complicated. The scientific method sets one up to try to discriminate as best as possible between truth and untruth. There is a very important question and one that I've talked about previously in other contexts. What are your responsibilities within your profession and, as a professional, outside of it? There's a novel called *Mephisto* by Klaus Mann, which is about an actor who sold himself to the Nazis to progress his career. After performing as Mephisto in Goethe's play *Faust*, someone comes and accuses him and says ‘You sold your soul to the Nazis’. He replies ‘Leave me alone, I'm just an actor’. The truth is that no one is just an actor or just a scientist. We all have responsibility to society. That is precisely why I spoke out against lockdown. What the Schneider quote is doing is something quite wicked. Because that quote is replacing social responsibility with social engineering. A scientist has social responsibility, but they should not be in a position to make decisions about what the public needs to hear and how they can manipulate the public into doing what they consider to be the right thing to do. I think you have to draw a very clear distinction between responsibility and power. A lot of mathematicians and modellers just say ‘I'm just a modeller, I just did the modelling’. That's not good enough either. I think, if like me, you felt that lockdowns would cause harm, you should have said so. I certainly felt obligated to speak out. But I didn't think it was my job to coerce the public to behave as I saw fit. What I wanted was constructive dialogue.

**There is one last question and that is a sensitive one. I saw that you were attacked from different angles. The faces that I saw who attacked you were White men. I thought that in any other context, everybody would shout ‘racism’ or ‘sexism’. In this context, I didn't hear a thing.**

It wasn't convenient for them. I don't particularly think that the people who were hurling abuse at me were actually and necessarily racist or sexist. What matters more to people, in this field anyway, is whether you are playing the game. I don't think that the people who were trying to shut us down were doing so because I was a woman. That wasn't their first concern; instead, it was to say ‘How dare you disagree with us?’. You could ask the question, had I been a White male, would they have treated me with the same level of disdain? Certainly in America, there are White males who were also treated very poorly. So I think what we saw was a manifestation of tribal behaviour rather than overt racism or sexism.
